# The Inquiry and after

**Published:** 1916-04-29

**Authors:** 


### NATIONAL INSURANCE ACT DEVELOPMENTS.

### The Inquiry and After.

We learn from reliable sources that the Committee of Inquiry appointed by the Treasury at the end of January last "to consider and report upon any amendments in the financial scheme of the National Insurance Acts which experience of the administration of sickness, disablement, and maternity benefits may suggest as desirable," have already made considerable progress in investigating the matters that fall within the scope of their reference. They have also examined in some detail the various suggestions that have been put forward for the simplification of the administration of the Act. The Committee are shortly to commence hearing witnesses from approved societies, and it is anticipated that when this evidence has been heard they will be in a position to frame their report. We understand that suggestions have been made, as the result of experience, which would have the effect of materially reducing the number of special classes with which the present administration is overburdened. Experience has shown, for instance, how little advantage has been taken of the provision by those entitled to become voluntary contributors. Again, the special rates of contribution for members of the mercantile marine, and for those whose employers guarantee the payment of full wages for a period of six weeks of sickness in a year, are excrescences of the system which entail labour, in connection not only with the account-keeping, but also in the actual administration of benefits, out of all proportion to the advantages conferred upon the classes of persons for whom these special provisions were made.

The various options allowed to insured women upon marriage are not understood. They are too complicated and technical, and it has been suggested that the Acts should be so amended as to require insured women upon marriage to relinquish insurance in consideration of a payment in the nature of a cash surrender value. Due provision would be made for the payment of employers' contributions should they continue or subsequently return to work, and benefits corresponding to those now given to exempted persons would be provided in return for such contributions.

These are all matters upon which it is clear that saving of expense of administration could be secured by simplification of administrative detail. It is, however, evident that if such simplification is to be made there must be legislative amendment of the present Acts. At the moment the special benefits to which reference has been made can be claimed by virtue of the Acts, and the rights and privileges cannot be abrogated by the Insurance Commissioners, nor by Order in Council, nor by any means other than an amendment of the National Insurance Acts.

Herein lies the difficulty of effecting the changes which may be shown conclusively by the Committee of Inquiry to be desirable. Unless the report of the Committee, when it is presented, is unanimous in its recommendations, and even, perhaps, in the event of unanimity being obtained, there is still the possibility that when the amendment Bill is introduced into Parliament, champions of the rights of the special classes may arise and endeavour to wreck the whole scheme of simplification unless their special pleas on behalf of restricted classes are met. It was, in fact, in this way that much of the present complication was introduced. As the amendments to be put forward will be the results of experience, the Government should be in a strong position to resist the temptation to weaken the effect of any strong action that may be contemplated. The warning is, however, none the less necessary because it is apparent that the introduction of an amendment Bill will provide an opportunity for the discussion of health insurance matters generally, in a way that has not been available in the House of Commons for some time past.

Another reason for apprehending difficulty in giving effect to the recommendations of the Committee of Inquiry arises from the fact that by the terms of reference they are restricted to the consideration of matters connected with the benefits administered by approved societies. Equally important matters are now pressing for attention in regard to the benefits that are administered through insurance committees—namely, medical and sanatorium benefits. It is hardly possible to conceive that an amendment Bill of the character that is anticipated as the result of the deliberations of the Committee of Inquiry would be allowed to go forward without incorporating provisions designed to improve the administration of medical benefit, and to put into a proper state of finance the present parlous condition of the sanatorium benefit funds.

If, then, these matters are to be incorporated in the amendment Bill, should not a further inquiry be instituted without delay, so that the provisions put forward under these headings should have at least the same support as those relating to sickness, disablement, and maternity benefits?

### Another Inquiry Required.

In our opinion such an inquiry is urgently required. The matters that would have to be considered are largely financial. Take, for instance, the question of medical benefit. Since the controversy between the medical profession and Mr. Lloyd George was settled in 1912 the whole economic situation has been changed. But in spite of this the allowance to the doctors *per capita* for attendance upon insured persons remains unaltered. On account of the increase in the price of drugs it is, indeed, probable that panel practitioners will find the amount available for their services actually reduced.

Moreover, the removal for the time being of large numbers of the most healthy men from the lists of panel patients on account of their having joined the Colours has not only reduced the number of patients, thereby restricting the amounts which the doctors will receive, but the mere fact that it is the most healthy section which has been withdrawn has altered the proportion of the amount of attention to be given. Again, as many of these same healthy men come back to civil life seriously incapacitated and are immediately replaced upon the insurance funds and require medical attention, which apart from the war would not have been the case; it is at once apparent that some alteration in the present basis will have to be adopted. The doctors are now bound by agreements which will not come up for revision until the end of the present year, and although advantage might be taken of this fact to defer the consideration of any alteration in the terms until the time arrives for terminating such agreement, it would probably be the wisest course to consider now, well in advance of such time, the various possible ways in which the situation may be relieved.

Reorganisation of Sanatorium Benefit Funds.

With regard to sanatorium benefit our columns have shown week after week how unsatisfactory a condition the administration of this benefit has fallen into. It is not entirely, even if it is primarily, due to lax administration on the part of certain Insurance Committees that they now find it impossible with the funds at their disposal to dispense the treatment that is promised under the Insurance Acts. Tuberculosis is not prevalent with the same intensity per unit of population throughout the areas of the different committees, and more effective means are required than those now available for readjusting the financial cost of the treatment as between the various committees.

A single central administrative council has much to recommend it, but on the other hand there is much that may be urged against centralised control which may, and often does, lack the power of adaptation to local needs.

In these circumstances the appointment of a small but strong Committee to inquire into and report upon the amendments in the administration of medical and sanatorium benefits which experience has shown to be desirable is a matter which should be pressed upon the attention of the Treasury.

